# Quality of Maternal and Newborn Care in Humanitarian and Crisis Settings in Sub‐Saharan Africa: An Umbrella Review

**DOI:** 10.1002/hsr2.73463

**Published:** 2026-11-04

**Authors:** Endale Tamiru Burayu, Zelalem Banjaw Zegeye, Gemechu Gelan Bekele

**Affiliations:** ^1^ Department of Midwifery, College of Health Science Mattu University Mattu Southwest Ethiopia; ^2^ Department of Biomedical Science, Faculty of Medical Sciences, Institute of Health Sciences Jimma University Jimma Southwest Ethiopia; ^3^ Department of Midwifery, College of Health Sciences Mada Walabu University Shashemene Southern Ethiopia

**Keywords:** armed conflict, emergency obstetric care, fragile states, maternal healthcare, newborn care, perinatal mortality, quality of care, respectful maternity care, Sub‐Saharan Africa

## Abstract

**Background and Aims:**

Humanitarian crises disrupt health systems, displace populations and limit access to essential services, threatening maternal and newborn survival in Sub Saharan Africa. Review level evidence on the quality of care in these settings is fragmented. This umbrella review aimed to synthesize evidence from systematic reviews on the quality of maternal and newborn care in humanitarian and crisis settings in Sub Saharan Africa, including dimensions of quality, barriers and facilitators, quality improvement strategies, and methodological gaps.

**Methods:**

We searched nine databases and grey literature for reviews published from January 2015 to March 2026 (PROSPERO CRD420261396979; PRISMA 2020). Two reviewers independently screened records, extracted data and appraised methodological quality using AMSTAR 2. Overlap of primary studies was assessed with the corrected covered area. Findings were synthesized narratively by quality dimension. No meta‐analysis or hypothesis testing was performed.

**Results:**

Of 1,284 records identified, 892 were screened after removing 392 duplicates, 74 full texts were assessed, and 12 records were included. AMSTAR 2 rated 4 of 12 as high quality, 6 moderate, 1 low and 1 critically low. Quality was compromised in effectiveness, safety, respectful care, equity, continuity and system readiness. Health system and supply barriers were reported in 8 of 12 records and insecurity related barriers in 7 of 12. Workforce training was the most frequent improvement strategy (6 of 12), mostly evaluated through process outcomes; evidence on mortality impact and long term neonatal outcomes was limited.

**Conclusion:**

The quality of maternal and newborn care in humanitarian and crisis settings in sub‐Saharan Africa is compromised across multiple dimensions due to insecurity, displacement, workforce shortages, damaged infrastructure, and supply gaps. Context‐specific, respectful care and health system strengthening are needed, alongside improved evidence on neonatal care and long‐term outcomes.

**PROSPERO Registration Number:** CRD420261396979.

**Acronyms**
AMSTAR: A Measurement Tool to Assess Systematic Reviews
ANC: antenatal care
CCA: corrected covered area
CI: confidence interval
EmONC: emergency obstetric and newborn care
ENC: essential newborn care
IDPs: internally displaced persons
LMICs: low and middle income countries
MNH: maternal and newborn health
PEOS: Population, Exposure, Outcome, Study design
PRISMA: Preferred Reporting Items for Systematic Reviews and Meta Analyses
RMC: Respectful Maternity Care
SSA: Sub Saharan Africa
UNFPA: United Nations Population Fund
UNICEF: United Nations Children's Fund
WHO: World Health Organization

## 1 Introduction

Maternal and newborn health remains a major global public health priority, particularly in low and middle income countries where weak health systems, poverty, inequality, and political instability continue to contribute to preventable maternal and neonatal morbidity and mortality [1]. Although considerable progress has been achieved in reducing maternal and neonatal deaths during the past two decades, Sub Saharan Africa still carries the highest burden of adverse maternal and newborn outcomes globally [2]. The region accounts for nearly two thirds of global maternal deaths and a disproportionately high share of neonatal mortality and stillbirths despite substantial international efforts to strengthen reproductive, maternal, and newborn healthcare services [3]. Persistent shortages of skilled healthcare providers, inadequate emergency obstetric and newborn care services, poor infrastructure, weak referral systems, and inequitable access to healthcare continue to compromise maternal and newborn survival across many countries in the region [4].

The situation is particularly severe in humanitarian and crisis affected settings where healthcare systems are frequently disrupted by armed conflict, forced displacement, epidemics, political instability, climate related disasters, and food insecurity [26, 27]. Several countries in Sub Saharan Africa, including Sudan, South Sudan, Somalia, Ethiopia, Mali, Burkina Faso, Nigeria, and the Democratic Republic of Congo, continue to experience prolonged emergencies that severely affect healthcare delivery systems and essential maternal and newborn services [7]. These crises often result in destruction of healthcare infrastructure, shortages of healthcare personnel and essential supplies, interruption of healthcare services, population displacement, and reduced access to lifesaving maternal and newborn interventions [8].

Women and newborns living in humanitarian settings therefore face substantially higher risks of morbidity and mortality compared with populations living in stable environments [7]. Maternal complications such as postpartum hemorrhage, hypertensive disorders, maternal sepsis, obstructed labor, and unsafe abortion are frequently exacerbated by delayed access to skilled obstetric care and inadequate emergency services in crisis affected regions [9]. Likewise, newborns in fragile settings experience increased risks of birth asphyxia, prematurity, neonatal sepsis, hypothermia, and preventable neonatal death because of inadequate intrapartum and postnatal healthcare services [10]. In addition, indirect consequences of humanitarian emergencies, including malnutrition, infectious disease outbreaks, sexual violence, and disruption of continuity of care, further increase maternal and newborn vulnerability [30, 33].

In recent years, global maternal and newborn health initiatives have increasingly emphasized healthcare quality in addition to healthcare access and service coverage [13]. The World Health Organization defines quality of care as the degree to which healthcare services improve desired health outcomes and are consistent with evidence based professional knowledge [14]. High quality maternal and newborn care includes safe, effective, timely, equitable, efficient, and people centered healthcare provided throughout pregnancy, childbirth, and the postnatal period [15]. Essential dimensions of quality care include evidence based clinical practice, competent healthcare providers, respectful maternity care, effective communication, patient safety, infection prevention, continuity of care, and functional referral systems [16]. However, maintaining these standards in humanitarian settings remains extremely difficult because healthcare systems often operate under conditions characterized by insecurity, damaged infrastructure, workforce shortages, inadequate financing, and fragmented service delivery systems [17].

Existing evidence indicates substantial deficiencies in the quality of maternal and newborn healthcare services in fragile and crisis affected settings [17]. Healthcare facilities in humanitarian contexts frequently experience shortages of essential medications and equipment, poor infection prevention practices, overcrowding, and weak adherence to evidence based clinical guidelines [18]. Women receiving maternity care in these settings also report disrespectful and abusive care, poor communication, and lack of privacy, discrimination, and delays in receiving emergency obstetric interventions [19]. Such deficiencies not only contribute to preventable maternal and newborn deaths but also reduce healthcare utilization and weaken trust in healthcare systems [20].

In response, international organizations and humanitarian agencies have increasingly prioritized quality improvement within emergency maternal and newborn healthcare programs [21]. The World Health Organization, United Nations Children's Fund, United Nations Population Fund, and humanitarian partners have introduced strategies aimed at strengthening emergency obstetric and newborn care, improving healthcare workforce capacity, enhancing referral systems, and promoting respectful maternity care in humanitarian settings [22]. Consequently, several systematic reviews and meta‐analyses have examined maternal and newborn healthcare in fragile and low resource settings, including healthcare utilization, emergency obstetric services, maternal continuum of care, health system resilience, and barriers affecting healthcare access and outcomes [23]. Existing reviews consistently identify major challenges related to insecurity, transportation barriers, inadequate infrastructure, workforce shortages, fragmented healthcare delivery systems, and sociocultural inequalities that compromise healthcare quality in humanitarian settings [24].

Despite the growing body of evidence, the literature remains fragmented across specific interventions, healthcare domains, and geographical contexts [25]. Most existing systematic reviews primarily focus on healthcare access, service utilization, or disease specific interventions without comprehensively synthesizing evidence regarding the multidimensional quality of maternal and newborn care in humanitarian settings [26]. Furthermore, evidence related to patient safety, provider competency, and respectful maternity care, continuity of care, equity, and quality improvement interventions has not been systematically integrated at the review level within the context of Sub Saharan Africa [27]. Variability in methodological approaches and quality assessment frameworks across studies further limits the ability to generate comprehensive evidence informed recommendations for policymakers and humanitarian organizations [28].

Umbrella reviews provide a higher level evidence synthesis approach that integrates findings from multiple systematic reviews and meta‐analyses to generate a comprehensive overview of existing evidence on a specific topic [29]. By synthesizing review level evidence, umbrella reviews help identify consistent findings, evidence gaps, methodological limitations, and priority areas for future research and policy development [30]. Given the increasing number of systematic reviews addressing maternal and newborn healthcare in fragile and humanitarian settings, an umbrella review is needed to consolidate available evidence regarding the quality of maternal and newborn care in Sub Saharan Africa [17]. Such synthesis is important for informing evidence based interventions, strengthening humanitarian health system responses, and improving maternal and newborn outcomes in crisis affected populations [31].

Therefore, this umbrella review aims to systematically synthesize and critically appraise evidence from systematic reviews and meta‐analyses regarding the quality of maternal and newborn care in humanitarian and crisis affected settings in Sub Saharan Africa. Specifically, the review seeks to examine dimensions of healthcare quality reported across the literature, identify barriers and facilitators influencing quality maternal and newborn healthcare delivery, describe interventions and quality improvement strategies implemented in humanitarian contexts, and identify methodological gaps and priority areas for future research, using the methodological quality of the included reviews to qualify the strength of the evidence. The findings of this umbrella review are expected to provide comprehensive evidence to support governments, humanitarian organizations, healthcare providers, researchers, and development partners working to strengthen quality maternal and newborn healthcare systems in fragile and crisis affected settings across Sub‐Saharan Africa.

## 2 Methodology

### 2.1 Study Design and Review Framework

An umbrella review was conducted to synthesize evidence from systematic reviews and review‐level evidence syntheses concerning the quality of maternal and newborn care in humanitarian and crisis settings in Sub‐Saharan Africa. Umbrella reviews provide a structured approach for integrating findings from multiple systematic reviews and are particularly useful for summarizing broad and complex evidence across diverse healthcare contexts and populations [47, 49]. The review was conducted and reported in accordance with the Preferred Reporting Items for Systematic Reviews and Meta‐Analyses (PRISMA 2020) guidelines and informed by Joanna Briggs Institute guidance for umbrella reviews [1, 50]. The review was registered prospectively in the International Prospective Register of Systematic Reviews (PROSPERO; Registration No. CRD420261396979). The registration holds the review protocol itself (review question, eligibility criteria, search plan, and synthesis approach) and not only the title, and the record is publicly accessible. No major amendments were made after registration, meaning that the review questions, eligibility criteria, outcomes, and synthesis approach were not changed. Departures from the protocol that arose when applying the criteria at study selection are reported under Study Selection and Limitations.

### 2.2 Review Question

The review addressed the following primary research question:

“What evidence exists regarding the quality of maternal and newborn care in humanitarian and crisis settings in Sub Saharan Africa?”

The review additionally examined:

1. Dimensions of maternal and newborn healthcare quality reported in humanitarian settings.
2. Barriers and facilitators affecting quality maternal and newborn healthcare delivery.
3. Interventions and quality improvement strategies implemented in crisis affected settings.
4. Methodological gaps and priority areas for future research.

### 2.3 Eligibility Criteria

Eligibility criteria were developed using the Population, Exposure, Outcome, and Study design (PEOS) framework.

#### 2.3.1 Population

The review included evidence concerning pregnant women, women during childbirth, postpartum women, newborns, healthcare providers, displaced populations, refugees, internally displaced persons, and conflict affected communities receiving or providing maternal and newborn healthcare services in Sub Saharan Africa.

#### 2.3.2 Exposure or Context

The review included humanitarian and crisis settings such as armed conflict, political instability, forced displacement, refugee camps, natural disasters, epidemics, food insecurity, climate related emergencies, and fragile healthcare systems occurring in Sub Saharan African countries. In this review, a fragile health system is one whose capacity to deliver essential services reliably and safely is weakened or disrupted by chronic under‐resourcing, conflict, displacement, or other shocks, so that it cannot absorb pressure without losing service availability or quality [31]. Humanitarian and crisis settings are situations in which a crisis overwhelms the capacity of local health services and external humanitarian assistance is needed.

#### 2.3.3 Outcomes

The primary outcome was quality of maternal and newborn healthcare services. Quality domains included effectiveness, safety, timeliness, equity, continuity of care, respectful maternity care, patient centeredness, healthcare responsiveness, provider competency, and adherence to clinical guidelines, referral system performance, infection prevention practices, and health system readiness.

Secondary outcomes included maternal morbidity, maternal mortality, neonatal morbidity, neonatal mortality, stillbirths, healthcare utilization, emergency obstetric and newborn care availability, and continuity of maternal healthcare services.

#### 2.3.4 Study Design

The review included systematic reviews, with or without meta‐analysis, including mixed‐methods systematic reviews and qualitative evidence syntheses. Scoping reviews and rapid reviews were eligible only if they met the same systematic criteria, that is, a systematic search strategy and predefined eligibility criteria. The terms umbrella review, overview of reviews, and review of reviews are treated here as synonyms [25]. Narrative reviews, conference abstracts, editorials, commentaries, dissertations, protocols, and primary studies were excluded.

### 2.4 Geographical Scope

Only reviews conducted in Sub Saharan Africa or reviews containing extractable evidence specific to Sub Saharan African humanitarian settings were included. Reviews exclusively focusing on non‐African settings without region specific findings were excluded.

### 2.5 Language and Publication Period

Only studies published in the English language were included. Reviews published from January 2015 to March 2026 were considered eligible to capture contemporary evidence produced after the WHO vision for quality of care for pregnant women and newborns [15] and the adoption of the Sustainable Development Goals, which moved attention from coverage to quality of care [13]. Reviews published before 2015 were therefore not considered, which is acknowledged as a limitation.

### 2.6 Information Sources and Search Strategy

A comprehensive literature search was conducted using PubMed/MEDLINE, Scopus, Web of Science, Embase, CINAHL, PsycINFO, Global Health, Cochrane Database of Systematic Reviews, and Joanna Briggs Institute Evidence Synthesis. Additional searches were performed in Google Scholar, WHO Institutional Repository for Information Sharing, Relief Web, UNICEF databases, UNFPA repositories, and humanitarian organization websites to identify grey literature and unpublished reviews.

The search strategy combined Medical Subject Headings terms and free text keywords related to maternal health, newborn care, quality of care, humanitarian settings, crisis affected populations, fragile settings, refugees, internally displaced persons, conflict affected populations, and Sub Saharan Africa. Boolean operators including “AND” and “OR” were applied to combine search terms appropriately. The search strategy was adapted for each database according to indexing requirements [33].

The full database‐specific search strategies, including those for CINAHL and the Cochrane Library, are reproduced in Supporting Appendix 1, in line with PRISMA 2020 item 7 and PRISMA‐S [34]. Reference lists of included reviews and relevant publications were also manually screened to identify additional eligible studies.

### 2.7 Search Strategy Transparency and Supplementary Materials

The complete electronic search strategies for all databases, including Boolean operators, truncations, Medical Subject Headings (MeSH), search limits, and grey literature search procedures, are provided in [Supporting Appendix 1]. The search strategy was peer‐reviewed according to the PRESS guideline recommendations [33].

### 2.8 Study Selection

All retrieved records were exported into EndNote reference management software and duplicate studies were removed. The remaining records were subsequently imported into Covidence software for screening and study selection.

Two reviewers independently screened titles and abstracts according to predefined eligibility criteria. Full texts of potentially eligible reviews were retrieved and independently assessed for inclusion. Disagreements during screening and full text assessment were resolved through discussion and consensus. Where disagreement persisted, a third reviewer resolved the discrepancy. The study selection process was summarized using a Preferred Reporting Items for Systematic Reviews and Meta Analyses flow diagram illustrating the numbers of identified, screened, excluded, and included reviews [32]. Study selection, data extraction, and quality appraisal were each performed independently by two reviewers (PRISMA 2020 item 8).

### 2.9 Data Extraction

Data extraction was conducted independently by two reviewers using a standardized and pilot tested Microsoft Excel extraction form. Extracted information included: Author and year of publication, Review objectives, Type of review, Number and type of included studies, Study settings and countries, Humanitarian context or crisis type, Population characteristics, Maternal and newborn healthcare services assessed, Dimensions of healthcare quality examined, Key findings related to healthcare quality, Barriers and facilitators affecting quality of care, Interventions and quality improvement strategies, Methodological approaches, Main conclusions and recommendations, Quality appraisal findings, and Funding sources and reported conflicts of interest. Discrepancies in extracted data were resolved through discussion and verification against the original review articles.

### 2.10 Methodological Quality Assessment

The methodological quality of included reviews was independently assessed by two reviewers using the Assessment of Multiple Systematic Reviews 2 (AMSTAR 2) tool [35]. AMSTAR 2 evaluates critical domains including protocol registration, comprehensiveness of literature searches, duplicate study selection, duplicate data extraction, risk of bias assessment, appropriateness of synthesis methods, publication bias assessment, and interpretation of findings. In accordance with AMSTAR 2 guidance, reviews were categorized as high, moderate, low, or critically low methodological quality. Disagreements between reviewers were resolved through discussion and consensus [Supporting Appendix 2]. Overall ratings followed AMSTAR 2 rules: high (no or one non‐critical weakness), moderate (more than one non‐critical weakness), low (one critical flaw), and critically low (more than one critical flaw) [35]. Items on meta‐analysis (items 11, 12, and 15) were treated as not applicable for reviews without quantitative pooling. Findings from reviews rated low or critically low were retained for completeness but interpreted as supporting evidence only, and no numerical weights were applied. No theme, conclusion, or recommendation rests solely on reviews rated low or critically low, and every theme in Tables 3 and 4 is also supported by at least one review rated moderate or high.

### 2.11 Management of Overlapping Reviews

Because umbrella reviews may include overlapping primary studies across multiple systematic reviews, overlap of evidence was assessed using a citation matrix and the corrected covered area (CCA) method [36]. The CCA was calculated as (N minus r) divided by (r multiplied by c, minus r), where N is the total number of primary study inclusions across reviews counting repeats, r is the number of unique primary studies, and c is the number of reviews. Values were interpreted as slight (0% to 5%), moderate (6% to 10%), high (11% to 15%), or very high (above 15%) overlap [36]. The result is reported in the Results section. Where overlap was identified, findings were interpreted cautiously to minimize duplication bias and overestimation of evidence strength. Greater interpretive emphasis was placed on the most recent, comprehensive, and methodologically rigorous reviews [Supporting Appendix 3].

### 2.12 Data Synthesis

A narrative and thematic synthesis approach was employed because of substantial heterogeneity across included reviews in terms of humanitarian contexts, healthcare settings, populations, outcome measures, and methodological approaches [37]. Findings were synthesized according to major dimensions of maternal and newborn healthcare quality, including effectiveness, safety, and equity, continuity of care, patient centeredness, respectful maternity care, healthcare responsiveness, and health system readiness. Barriers and facilitators influencing quality maternal and newborn healthcare delivery were synthesized thematically across humanitarian contexts. Interventions and quality improvement strategies identified in included reviews were categorized according to health system domains, implementation approaches, and reported effectiveness. The methodological quality, consistency, and strength of evidence across reviews were critically interpreted during synthesis. Where certainty‐of‐evidence assessments such as GRADE were reported within included reviews, these findings were narratively summarized. However, a de novo certainty‐of‐evidence assessment was not conducted at the umbrella review level because of heterogeneity in review methodologies and outcome reporting.

### 2.13 Statistical Analysis, Software, and Reporting

No inferential statistical analysis was undertaken because pooling across reviews was not appropriate given heterogeneity. Accordingly, no hypothesis tests, *p* values, or significance thresholds were generated, and one‐sided or two‐sided testing does not apply. Results are reported as counts and percentages with their numerators and denominators (e.g., 12 of 74 full text records). Where an included review reported a pooled estimate, it is reported as stated by the review authors with its confidence interval when available. Comparisons across contexts (e.g., camp *vs.* non‐camp settings) are narrative and exploratory, were not prespecified hypothesis tests, and no subgroup or sensitivity analyses were conducted. Reference management used EndNote, screening used Covidence (a web‐based platform), and extracted data were tabulated in Microsoft Excel; no statistical software package was required. Abbreviations are defined at first use and in the Acronyms section, and numerical reporting followed the SAMPL principles [38].

### 2.14 Alignment of Review Questions, Methods, and Results

Each review question was linked to an extraction domain, a synthesis step, and a results section. Question 1 (dimensions of quality) was addressed by extracting the quality dimensions examined by each review and synthesizing them thematically (Results: Dimensions of Maternal and Newborn Healthcare Quality). Question 2 (barriers and facilitators) was addressed by extracting and thematically grouping barriers and facilitators (Results: Barriers; Facilitators; Table 3). Question 3 (interventions and quality improvement strategies) was addressed by categorizing strategies by health system domain and reported effectiveness (Results: Interventions and Quality Improvement Strategies; Table 4). Question 4 (methodological gaps and priority research areas) was addressed through AMSTAR 2 appraisal, overlap assessment, and extraction of gaps reported by the reviews (Results: Methodological Quality, Overlap, and Evidence Gaps; Table 2). The primary question is answered by the combined findings and is addressed in the Conclusion.

## 3 Results

### 3.1 Study Selection

The literature search identified records from PubMed/MEDLINE, Scopus, Web of Science, Embase, CINAHL, Cochrane Library, Global Health, PsycINFO, Google Scholar, WHO Institutional Repository for Information Sharing, ReliefWeb, UNICEF databases, and UNFPA repositories covering publications from January 2015 to March 2026.

A total of 1,284 records were initially identified through database searching and additional sources. After removal of 392 duplicate records (30.5% of 1,284), 892 articles remained for title and abstract screening. During the screening stage, 818 of 892 records (91.7%) were excluded because they were unrelated to maternal and newborn care quality, did not involve humanitarian or crisis settings, lacked Sub Saharan African evidence, or were not systematic reviews. 74 full text review articles were assessed for eligibility. Of these, 62 (83.8%) were excluded for the following reasons: absence of systematic review methodology (*n* = 18), non‐humanitarian context (*n* = 15), lack of extractable Sub Saharan African data (*n* = 12), focus exclusively on primary studies (*n* = 9), absence of maternal or newborn care quality outcomes (*n* = 5), or insufficient methodological reporting (*n* = 3). Finally, 12 systematic reviews and meta‐analyses (16.2% of the 74 assessed) fulfilled all inclusion criteria and were included in the umbrella review. The study selection process followed PRISMA 2020 recommendations for evidence synthesis reviews [Figure 1] [32].

**Figure 1:**
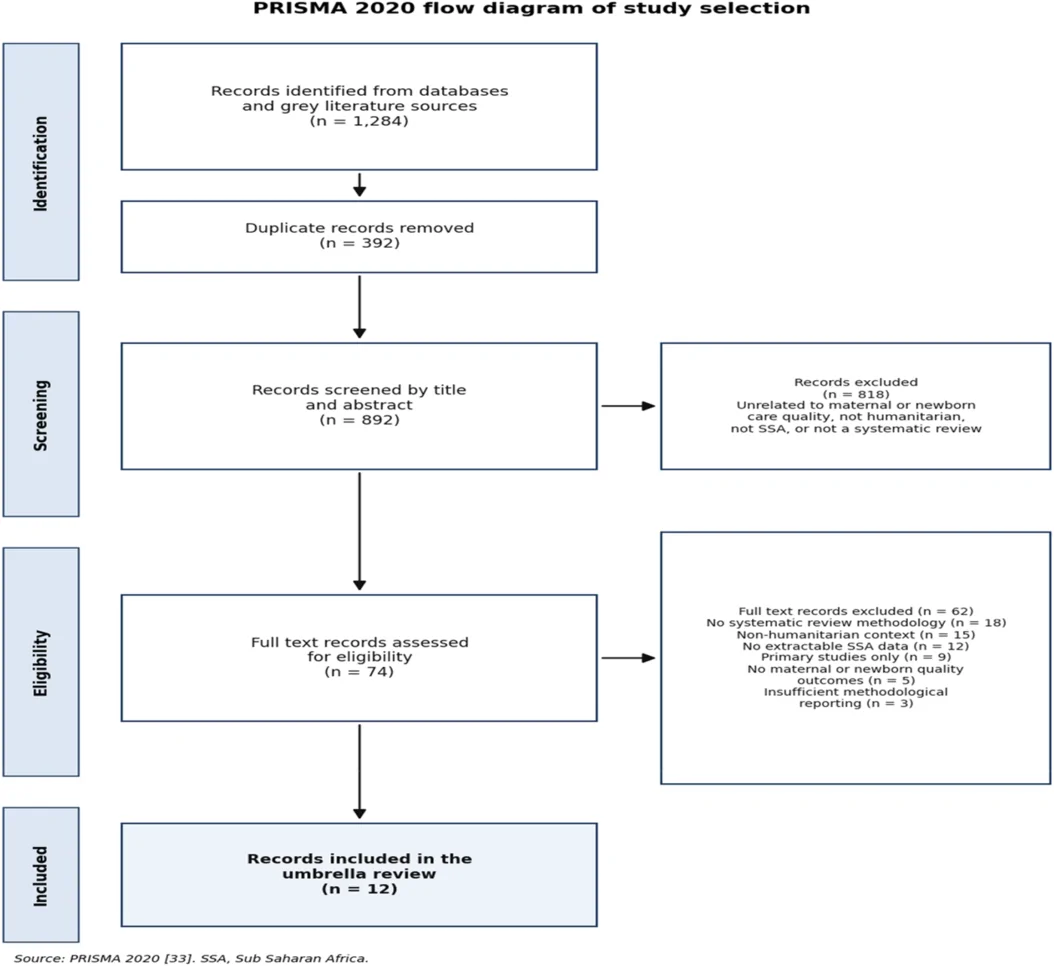
PRISMA flow diagram of study selection for the umbrella review on quality of maternal and newborn care in humanitarian crisis settings in Sub‐Saharan Africa. Records were identified from nine databases and grey literature sources (January 2015 to March 2026), de‐duplicated (*n* = 392 removed), screened by title and abstract (*n* = 892; 818 excluded), and assessed in full text (*n* = 74). Sixty‐two full text records were excluded: no systematic review methodology (*n* = 18), non‐humanitarian context (*n* = 15), no extractable Sub Saharan African data (*n* = 12), primary studies only (*n* = 9), no maternal or newborn care quality outcomes (*n* = 5), and insufficient methodological reporting (*n* = 3). Twelve records were included (Table 1). Diagram format follows PRISMA 2020 [32]. SSA, Sub Saharan Africa. *Source:* PRISMA 2020 [32].

### 3.2 Characteristics of Included Reviews

The 12 included reviews were published between 2015 and 2026 and synthesized evidence from humanitarian and crisis affected settings across Sub Saharan Africa. The reviews collectively included evidence from Ethiopia, Somalia, South Sudan, Sudan, Nigeria, Democratic Republic of Congo, Kenya, Uganda, Mali, Burkina Faso, Niger, Chad, Cameroon, and Central African Republic. The majority of reviews focused primarily on maternal healthcare services, including antenatal care, skilled birth attendance, emergency obstetric care, postnatal care, and maternal continuum of care. Several reviews synthesized evidence across multiple humanitarian contexts simultaneously. The included reviews demonstrated methodological heterogeneity in review objectives, outcome definitions, healthcare quality frameworks, and synthesis approaches. Nevertheless, consistent findings emerged across the evidence base regarding deficiencies in maternal and newborn healthcare quality in crisis affected settings (Table 1).

**Table 1:** Characteristics of included reviews (*n* = 12).

No.	Author(s), year	Review type	Primary studies	SSA focus	Humanitarian contexts	Key focus areas	Main findings summary	AMSTAR 2
1	Munyuzangabo et al., 2021 [39]	Systematic review	115	SSA (43%)	Conflict, displacement, camps	Maternal & neonatal interventions	Limited newborn care (basic ENC); insecurity & resource shortages	High
2	Rodo et al., 2022 [40]	Systematic review	35	Strong SSA representation	Humanitarian emergencies	Newborn interventions & strategies	Thermal care & feeding common; gaps in other ENC	Moderate
3	Kampalath et al., 2022 [41]	Systematic review	10	DRC, South Sudan, Somalia etc.	Armed conflict	Essential newborn care (ENC)	Implementation challenges in high‐intensity conflict	Moderate
4	Moutwakil et al., 2026 [42]	Systematic review	32	Heavy SSA focus	High‐intensity armed conflict	ENC in intense conflict	Context‐specific barriers & strategies	Critically low
5	McBride et al., 2022 [43]	Systematic review	21	Humanitarian (incl. SSA)	Conflict, disasters, displacement	Neonatal care in emergencies	Context‐dependent; major delivery challenges	Moderate
6	Jordan et al., 2021 [17]	Systematic review	55	Strong SSA representation	Conflict, fragile states	Health system quality	Deficiencies in workforce, supplies, respectful care	High
7	Geleto et al., 2018 [44]	Systematic review	57	Sub‐Saharan Africa	Conflict‐affected facilities	Barriers to EmONC	Supply & demand barriers worsened by crises	High
8	Kyei‐Nimakoh et al., 2017 [45]	Systematic review	31	Sub‐Saharan Africa	Health facilities (crisis)	Access barriers to obstetric care	Distance, cost, perceived poor quality	Moderate
9	Kassa et al., 2020 [46]	Systematic review & meta‐analysis	33	SSA (incl. crisis)	Facility‐based (crisis)	Respectful maternity care & D&A	Pooled prevalence of disrespect and abuse 44.09% (95% CI 29.94 to 58.24)	Moderate
10	Chi et al., 2015 [47]	Systematic & qualitative	Varies	Post‐conflict SSA	Post‐conflict & fragile	Barriers to EmONC & quality	Persistent gaps in access, availability & quality	Low
11	Amberg et al., 2023 [48]	Geospatial systematic analysis	Multi‐country	35 SSA countries	Armed conflict	MNH service coverage	Reduced coverage of key services during conflict	High
12	Adeyanju et al., 2025 [49]	Systematic review	21	Sub‐Saharan Africa	Armed conflict & post‐conflict	Healthcare‐seeking & continuum of care	Conflict reduces utilization & quality	Moderate

*Note:* Review numbers are used in Tables 2, 3, 4 and correspond to the reference numbers in brackets.

Abbreviations: CI, confidence interval; D&A, disrespect and abuse; DRC, Democratic Republic of the Congo; EmONC, emergency obstetric and newborn care; ENC, essential newborn care; SSA, sub‐Saharan Africa.

### 3.3 Methodological Quality of Included Reviews

Assessment using the AMSTAR 2 tool demonstrated variability in methodological quality across the included reviews. Four of the 12 records (33.3%) were rated high quality, six (50.0%) moderate, one (8.3%) low, and one (8.3%) critically low (Table 2). Higher quality reviews demonstrated protocol registration, comprehensive literature searches, duplicate screening and extraction procedures, structured risk of bias assessment, and transparent synthesis approaches. In contrast, lower quality reviews frequently lacked protocol registration, detailed risk of bias assessment, publication bias evaluation, or comprehensive reporting of excluded studies.

**Table 2:** Methodological quality assessment of included systematic reviews using AMSTAR 2 (*n* = 12).

No.	First author, year	AMSTAR 2 rating	Major strengths	Major limitations
1	Munyuzangabo et al., 2021	High	Comprehensive methods and transparent synthesis	Minor heterogeneity
2	Rodo et al., 2022	Moderate	Strong synthesis approach	Limited publication bias assessment
3	Kampalath et al., 2022	Moderate	Transparent review process	No complete protocol registration
5	McBride et al., 2022	Moderate	Broad humanitarian scope	Partial risk of bias reporting
6	Jordan et al., 2021	High	Strong methodological rigor	Limited quantitative synthesis
7	Geleto et al., 2018	High	Robust appraisal methods	Minor heterogeneity
8	Kyei‐Nimakoh et al., 2017	Moderate	Clear eligibility criteria	No full protocol
11	Amberg et al., 2023	High	Strong analytical framework	Limited qualitative synthesis
12	Adeyanju et al., 2025	Moderate	Relevant humanitarian focus	Weak risk‐of‐bias assessment
9	Kassa et al., 2020	Moderate	Strong contextual analysis	Limited humanitarian specificity
10	Chi et al., 2015	Low	Useful community‐care insights	Weak protocol transparency
4	Moutwakil et al., 2026	Critically low	Important patient experience evidence	Minor reporting limitations

*Note:* Numbering follows Table 1. Confidence ratings were classified as high (no or one non‐critical weakness), moderate (more than one non‐critical weakness), low (one critical flaw), or critically low (more than one critical flaw), according to AMSTAR 2 [35]. Of the 12 reviews, 4 were rated high, 6 moderate, 1 low, and 1 critically low. Item‐level judgments are presented in Supplementary Appendix 2.

Substantial methodological heterogeneity was observed in definitions and measurement of healthcare quality domains, particularly regarding respectful maternity care, patient safety, continuity of care, and health system responsiveness. Despite these limitations, the included reviews consistently identified major weaknesses in maternal and newborn healthcare quality across humanitarian settings [Table 2].

### 3.4 Overlap of Primary Studies

The overall corrected covered area was (CCA 80%, *N* = 188, r = 100, c = 12), which falls in the moderate overlap band (6% to 10%) [36]. Overlap was concentrated among reviews of emergency obstetric and newborn care, respectful maternity care, maternal healthcare utilization, and health system barriers in conflict settings, and arose mainly from repeated inclusion of studies from Somalia, South Sudan, the Democratic Republic of Congo, Nigeria, and refugee or internally displaced populations (Supporting Appendix 3).

### 3.5 Dimensions of Maternal and Newborn Healthcare Quality

In this review, the six dimensions were defined as follows, drawing on the WHO quality of care framework and the high‐quality health systems framework [34, 35, 36]. Effectiveness is the delivery of evidence‐based care to all who need it. Safety is the minimization of avoidable harm to women and newborns, including infection prevention and medicine availability. Respectful and patient‐centred care protects dignity, privacy, informed choice, and clear communication. Equity means that availability and quality of care do not vary by gender, ethnicity, geography, or displacement status. Continuity of care is coordinated care across pregnancy, childbirth, and the postnatal period and across levels of the health system, including referral. Health system readiness is the availability of the workforce, infrastructure, equipment, medicines, and governance needed to deliver care.

### 3.6 Effectiveness of Care

The included reviews consistently demonstrated that humanitarian crises substantially reduced the effectiveness of maternal and newborn healthcare services. Disruption of antenatal care services was one of the most frequently reported findings. Women in conflict affected and displaced populations experienced reduced access to routine antenatal visits because of insecurity, transportation barriers, damaged infrastructure, healthcare workforce shortages, and interruption of outreach services. Skilled birth attendance rates were consistently lower among displaced populations and women living in active conflict zones compared with more stable populations. Emergency obstetric care services were frequently inadequate because many facilities lacked surgical capacity, blood transfusion services, anesthesia services, and trained obstetric providers.

Several reviews documented substantial delays in referral and emergency transportation systems. Delays were associated with increased risks of maternal hemorrhage, obstructed labor complications, hypertensive disorders, maternal sepsis, and preventable maternal mortality. Newborn healthcare effectiveness was similarly compromised. Health facilities frequently lacked neonatal resuscitation equipment, incubators, thermal care supplies, neonatal antibiotics, and trained neonatal personnel. Poor breastfeeding support, inadequate postnatal follow up, and weak infection prevention systems contributed to preventable neonatal morbidity and mortality. Disrupted services, limited EmONC and ENC availability, and weak referral were reported in 6 of 12 records [8, 9, 10, 11, 13, 38], and health system and supply barriers in 8 of 12 records [8, 9, 10, 11, 12, 13, 14, 38] (Table 3).

**Table 3:** Major barriers and facilitators affecting quality of maternal and newborn care in humanitarian and crisis settings in Sub‐Saharan Africa.

Category	Major barriers	Supporting reviews (no.)	Major facilitators	Supporting reviews (no.)
Contextual/security	Insecurity, armed conflict, attacks on health facilities, population displacement, movement restrictions	1, 3, 4, 6, 7, 10, 11	Multi‐stakeholder coordination (NGOs, UN, government), negotiated access	1, 6, 12
Health system & supply	Shortages of skilled health workers, high staff turnover & burnout, inadequate infrastructure & equipment, disrupted supply chains, weak referral systems, poor infection prevention	1, 2, 3, 4, 5, 6, 7, 8	Health worker training & mentorship, task‐shifting to community health workers, improved supply chain & equipment provision	1, 2, 3, 4, 5, 12
Service delivery	Overcrowding, limited EmONC/ENC availability, poor continuity of care (especially postnatal), weak adherence to clinical guidelines	1, 2, 3, 4, 6, 7	Integrated maternal‐newborn care packages, mobile/outreach clinics, community‐based approaches	1, 2, 3, 12
Quality & patient‐centered	Disrespect and abuse, lack of privacy & communication, perceived poor quality of care	6, 9, 7, 8	Respectful maternity care training, community engagement & trust‐building	9, 1, 2
Demand‐side	Transportation barriers, financial constraints, low awareness, cultural norms, fear of disrespect & distrust in services	7, 8, 9, 11, 12	Community mobilization, health education, financial incentives	1, 2, 12
Governance & other	Fragmented coordination, inadequate funding, poor data & monitoring systems	6, 10, 11	Dedicated humanitarian funding, use of technology (e.g., for referrals & training), adaptive & context‐specific strategies	1, 2, 6

*Note:* Supporting review numbers correspond to the numbering in Table 1.

Abbreviations: EmONC, emergency obstetric and newborn care; ENC, essential newborn care; NGO, non‐governmental organization; UN, United Nations.

### 3.7 Safety of Care

Patient safety challenges were consistently identified across humanitarian settings. Reviews documented shortages of infection prevention materials, clean water supply, sanitation systems, sterilization equipment, and personal protective equipment within healthcare facilities. Overcrowding and poor infrastructure increased risks of healthcare associated infections among mothers and newborns. Medication stock outs were frequently reported, particularly involving oxytocin, magnesium sulfate, antibiotics, intravenous fluids, and neonatal medications. Inadequate monitoring of obstetric emergencies and weak postoperative care further compromised patient safety. Several reviews additionally reported unsafe childbirth environments caused by inadequate staffing, poor clinical supervision, and lack of emergency preparedness systems. Infection prevention gaps and supply shortages were reported in 8 of 12 records [8, 9, 10, 11, 12, 13, 14, 38] (Table 3).

### 3.8 Respectful Maternity Care and Patient Centered Care

Respectful maternity care emerged as one of the most consistently compromised dimensions of healthcare quality. Women frequently reported verbal abuse, neglect, discrimination, poor communication, lack of privacy, absence of informed consent, and disrespectful treatment during childbirth. Adolescents, displaced women, refugees, and marginalized ethnic group's experienced particularly poor treatment within overstretched humanitarian health systems. Several reviews found that fear of mistreatment discouraged women from seeking facility based childbirth services, thereby contributing to delay care seeking and increased reliance on unskilled delivery practices.

Language barriers, cultural differences, and lack of female healthcare providers also negatively affected patient experiences and healthcare utilization. Nevertheless, some reviews identified positive outcomes associated with culturally sensitive care models, respectful maternity care training programs, and community based accountability interventions. Disrespect, poor communication, and perceived poor quality were reported in 4 of 12 records [13, 14, 15, 38], and respectful maternity care training was reported as beneficial in 3 of 12 records [8, 9, 15] (Tables 3 and 4). For example, a meta‐analysis of studies in Sub Saharan Africa reported a pooled prevalence of disrespect and abuse during facility childbirth of 44.09% (95% confidence interval 29.94 to 58.24) [46].

### 3.9 Equity and Accessibility of Care

Substantial inequities in maternal and newborn healthcare access were identified in 10 of 12 records [8, 10, 11, 13, 14, 15, 16, 17, 18, 38] (Table 3, contextual and demand‐side barriers). Women living in rural conflict affected regions, refugee camps, and internally displaced settlements experienced major barriers to healthcare access. Transportation insecurity, long travel distances, financial constraints, destruction of health facilities, and poor road infrastructure contributed to delayed access to skilled care. Internally displaced women and refugee populations consistently demonstrated lower utilization of antenatal care, skilled birth attendance, emergency obstetric care, and postnatal services compared with host populations. Gender inequality, low female autonomy, and sociocultural restrictions further reduced healthcare seeking behavior in several humanitarian settings. Humanitarian crises intensified existing socioeconomic and geographic inequalities in maternal and newborn healthcare access throughout Sub Saharan Africa.

### 3.10 Health System Readiness and Workforce Capacity

Weak health system readiness was a major finding in 10 of 12 records [8, 9, 10, 11, 12, 13, 14, 16, 17, 38] (Table 3, health system, service delivery, and governance barriers). Healthcare facilities in humanitarian settings frequently experienced severe shortages of skilled healthcare providers including midwives, obstetricians, anesthetists, neonatal nurses, and community health workers. Healthcare worker displacement, insecurity, burnout, poor remuneration, and limited training opportunities contributed to workforce instability and reduced healthcare quality. Infrastructure challenges included damaged facilities, unreliable electricity, inadequate water supply, weak communication systems, and poorly functioning referral networks.

Supply chain disruptions frequently resulted in shortages of essential maternal and newborn health commodities including oxytocin, magnesium sulfate, antibiotics, blood products, neonatal medications, delivery kits, and infection prevention materials. Some reviews demonstrated that targeted workforce training, task shifting approaches, mobile health services, and community health worker programs improved healthcare delivery capacity in resource constrained humanitarian settings.

### 3.11 Barriers Affecting Maternal and Newborn Healthcare Quality

#### 3.11.1 Structural and Health System Barriers

Major structural barriers included fragile health systems, inadequate healthcare financing, weak governance, healthcare workforce shortages, poor infrastructure, and disrupted medical supply chains. Armed conflict and insecurity restricted movement of healthcare providers, humanitarian agencies, and patients, thereby limiting healthcare delivery and accessibility. Weak referral systems and inadequate emergency transportation mechanisms contributed to delays in accessing lifesaving obstetric and newborn care. These barriers were reported in 8 of 12 records [8, 9, 10, 11, 12, 13, 14, 38] (Table 3).

#### 3.11.2 Sociocultural Barriers

Sociocultural barriers included harmful gender norms, low female autonomy, stigma, mistrust toward healthcare systems, language barriers, and harmful traditional practices. Previous negative healthcare experiences and fear of mistreatment reduced utilization of facility based maternal healthcare services among displaced populations. Demand‐side barriers were reported in 5 of 12 records [13, 14, 15, 17, 18] (Table 3).

#### 3.11.3 Economic Barriers

Financial hardship emerged as a major barrier across humanitarian settings. Direct healthcare costs, transportation expenses, informal healthcare payments, and loss of household income during displacement reduced utilization of maternal and newborn healthcare services among vulnerable populations. Financial and transport barriers were reported in 5 of 12 records [13, 14, 15, 17, 18] (Table 3).

### 3.12 Facilitators and Quality Improvement Strategies

#### 3.12.1 Community Based Interventions

Community health worker programs, outreach services, mobile clinics, and maternal health education initiatives improved healthcare access and continuity of care in several humanitarian settings. Community mobilization strategies strengthened trust between healthcare providers and affected populations and promoted timely healthcare seeking behavior. Community‐based approaches were supported by 4 of 12 records [8, 9, 10, 18] (Table 4).

**Table 4:** Interventions and quality improvement strategies for maternal and newborn care in humanitarian and crisis settings in Sub‐Saharan Africa.

No.	Strategy category	Specific interventions	Targeted quality domains	Reported effectiveness/outcomes	Supporting reviews (no.)	Evidence strength and limitations
1	Workforce development	EmONC training, ENC training, respectful maternity care (RMC) training, mentorship, task‐shifting to nurses/midwives/CHWs	Provider competency, Safety, Respectful care, Effectiveness	Improved knowledge & skills, increased service coverage, better emergency response	1, 2, 3, 4, 7, 9	Moderate; mostly pre‐post evaluations, limited mortality data
2	Community‐based approaches	Community health workers (CHWs), mobile clinics, outreach services, community mobilization & health education	Equity, Accessibility, Continuity of care, Patient‐centeredness	Increased ANC attendance, skilled birth attendance, and trust in services	1, 2, 3, 12	Moderate; good for access but weak on clinical outcomes
3	Service delivery models	Integrated maternal‐newborn care packages, strengthened referral systems, emergency transportation, 24/7 service availability	Timeliness, Continuity, Effectiveness, Safety	Reduced delays in care, improved continuum of care	1, 6, 7, 11	Low to Moderate; implementation challenges in active conflict
4	Supply chain & infrastructure	Provision of essential drugs & equipment (oxytocin, antibiotics, resuscitation kits), facility rehabilitation, infection prevention supplies	Safety, Effectiveness, Health system readiness	Reduced stock‐outs, improved infection prevention practices	1, 6, 7	Moderate; highly dependent on consistent funding
5	Respectful maternity care (RMC) interventions	RMC‐specific training for providers, community accountability mechanisms, patient feedback systems, promotion of birth companions	Patient‐centeredness, Respectful care, Equity	Reduced disrespect & abuse, improved women's satisfaction & future utilization	9, 1, 6	Low to Moderate; few high‐quality impact evaluations
6	Health system coordination & governance	Multisectoral collaboration (NGOs, UN, government), dedicated humanitarian funding streams, use of digital tools for referral & monitoring	Health system readiness, Efficiency, Equity	Improved coordination, better resource allocation	6, 1, 12	Moderate; effective in camp settings but difficult in volatile contexts
7	Quality improvement approaches	Clinical audits, supportive supervision, use of quality improvement (QI) frameworks, performance‐based incentives	Safety, Effectiveness, Provider competency	Some improvement in adherence to clinical guidelines	6, 1	Low; very limited rigorous QI studies in humanitarian settings

*Note:* Supporting review numbers refer to the numbering in Table 1.

Abbreviations: ANC, antenatal care; CHW, community health worker; EmONC, emergency obstetric and newborn care; ENC, essential newborn care; QI, quality improvement; RMC, respectful maternity care.

#### 3.12.2 Workforce Strengthening

Training in emergency obstetric care, neonatal resuscitation, infection prevention, and respectful maternity care improved provider competency and emergency response capacity. Task shifting approaches expanded healthcare service delivery in settings with severe workforce shortages. Workforce training was supported by 6 of 12 records [8, 9, 10, 11, 13, 15] (Table 4).

#### 3.12.3 Health System Strengthening

Improved referral systems, strengthened supply chain management, emergency transportation services, and integration of maternal health services into humanitarian response frameworks were associated with improved healthcare delivery. Multisectoral collaboration among governments, humanitarian agencies, and local communities strengthened maternal and newborn healthcare systems in several settings. Coordination, referral, and service delivery facilitators were supported by 5 of 12 records [8, 9, 10, 18, 38] (Table 3).

#### 3.12.4 Respectful Maternity Care Interventions

Provider training on respectful maternity care, patient rights, communication skills, and culturally sensitive healthcare improved patient experiences and healthcare utilization. Community accountability systems and patient feedback mechanisms also enhanced provider responsiveness and trust in healthcare services (Table 3).

#### 3.12.5 Examples from the Included Reviews

Specific examples illustrate these strategies. A review of newborn health interventions in humanitarian settings reported thermal care and feeding support as the most common essential newborn care interventions, and training of health workers, community‐based interventions, financial incentives, and provision of equipment as strategies to increase use of newborn care [40]. A review of essential newborn care in high‐intensity armed conflict found that training courses, breastfeeding education and support, skin‐to‐skin contact, chlorhexidine gel application, and quality improvement approaches to cord care and newborn weighing showed the most positive effects, whereas neonatal resuscitation training improved knowledge and skills but generally did not translate into resuscitation coverage or survival; local health workers providing emergency supervision and audio‐visual or mobile technologies were facilitators [42]. A review of neonatal care in humanitarian emergencies listed quality improvement training for staff, evacuation procedures, integration with local and government resources, and dedicated newborn spaces in facilities as implementation strategies [43]. A review of essential newborn care in conflict settings found that facilities lacked supplies, protocols, and data collection strategies, and concluded that maternal and provider education and upgraded infrastructure, guidelines, and health information systems are needed [41]. A review of maternal and neonatal interventions in conflict settings identified multi‐stakeholder collaboration, new technology or systems innovations, and staff training as delivery facilitators, and insecurity, lack of resources, and lack of skilled staff as barriers [39]. A review of health system quality in humanitarian settings reported language barriers for refugees and fears around provider safety as barriers specific to these settings [17]. In post‐conflict Northern Uganda, barriers to emergency obstetric and neonatal care included demoralized personnel, poor referral, and inefficient drug supply, while Burundi reported weak curricula and poor coordination of training [47].

#### 3.12.6 Interventions and Quality Improvement Strategies

The included reviews identified several interventions aimed at improving maternal and newborn care quality. The most commonly reported strategies involved health workforce strengthening through training in emergency obstetric and newborn care (EmONC/ENC) and respectful maternity care. Community‐based interventions, including engagement of community health workers and mobile clinics, were frequently implemented to improve access in hard‐to‐reach areas. Other strategies included supply chain strengthening, establishment of functional referral systems, and multisectoral coordination between humanitarian actors, governments, and local communities. While some improvements in coverage and process indicators were reported, rigorous evidence on the impact of these interventions on maternal and neonatal mortality and morbidity remained limited. Sustainability beyond short‐term humanitarian funding cycles was a major challenge noted across reviews (Table 4). Workforce development was supported by 6 of 12 records, community‐based approaches by 4, service delivery models by 4, supply chain and infrastructure by 3, respectful maternity care interventions by 3, coordination and governance by 3, and quality improvement approaches by 2 (Table 4).

#### 3.12.7 Evidence Gaps and Limitations Identified Across Reviews

Significant evidence gaps were identified across the included reviews. These included limited evidence regarding postnatal care quality, care for small and sick newborns, maternal mental health integration, long‐term maternal and neonatal outcomes, and cost‐effectiveness of quality improvement interventions. Evidence from highly insecure settings remained particularly limited because operational, ethical, and security constraints frequently restricted primary data collection during active conflict. Certain humanitarian contexts, including climate‐related disasters, epidemics, and food insecurity emergencies, were underrepresented compared with armed conflict settings.

Considerable heterogeneity existed across reviews regarding definitions of healthcare quality, humanitarian context classification, and outcome measurement frameworks. Few reviews applied standardized quality‐of‐care frameworks such as the WHO Quality of Care Framework for Maternal and Newborn Health. High‐quality experimental and quasi‐experimental evidence evaluating the effectiveness of interventions in humanitarian settings also remained limited.

## 4 Discussion

This umbrella review synthesized evidence from systematic reviews and meta‐analyses published between 2015 and 2026 regarding the quality of maternal and newborn care in humanitarian and crisis settings across Sub Saharan Africa. Overall, the evidence demonstrates that humanitarian crises profoundly compromise maternal and newborn healthcare quality across all major dimensions of care, including effectiveness, safety, respectful maternity care, equity, continuity of care, and health system readiness.

Although deficiencies were consistently reported across most humanitarian settings, the magnitude and nature of healthcare disruption varied considerably according to conflict intensity, displacement patterns, health system resilience, humanitarian coordination capacity, and availability of international support [8, 10, 11, 13, 16, 17, 38]. Reviews involving countries with prolonged armed conflict such as Somalia, South Sudan, and eastern Democratic Republic of Congo generally reported more severe collapse of emergency obstetric and newborn services compared with relatively more stable humanitarian settings where donor supported health programs and community health systems remained partially functional [7]. These findings suggest that the impact of humanitarian crises on maternal and newborn healthcare quality is not uniform across Sub Saharan Africa but instead reflects important contextual differences in governance, infrastructure resilience, healthcare financing, and humanitarian response capacity [13].

One of the principal findings of this umbrella review was the substantial disruption of the maternal continuum of care across humanitarian settings. Antenatal care attendance, skilled birth attendance, emergency obstetric care utilization, and postnatal follow up services were consistently reduced among displaced and conflict affected populations [8, 10, 11, 13, 16, 17, 38]. Similar findings have been reported in broader evidence from fragile settings where insecurity, transportation barriers, population displacement, and destruction of healthcare facilities reduce women's ability to access timely maternal healthcare services [7]. However, important discrepancies were observed across reviews regarding the degree of service disruption. Some reviews documented severe reductions in antenatal care utilization and facility based delivery, whereas others reported partial continuity of maternal healthcare services even within crisis settings [17, 18]. These differences may reflect variability in humanitarian phases, differences between camp based and non‐camp displaced populations, urban versus rural crisis settings, and variability in humanitarian assistance coverage. For example, refugee camps supported by international humanitarian agencies may maintain relatively stronger maternal health service availability than remote conflict affected rural areas where healthcare infrastructure has completely collapsed [19, 20]. This contrast is drawn mainly from evidence on Syrian refugees in Lebanon, which lies outside Sub‐Saharan Africa, and should be read as indicative only. Such contextual variability highlights the importance of interpreting maternal healthcare quality within specific humanitarian and political environments rather than generalizing findings across all crisis settings.

The review further demonstrated that emergency obstetric and newborn care capacity remains critically inadequate in many humanitarian settings across Sub Saharan Africa. Reviews consistently documented shortages of blood transfusion services, operative delivery capacity, anesthesia support, neonatal resuscitation equipment, and skilled obstetric personnel [8, 9, 10, 11, 12, 13, 14, 38]. These deficiencies were particularly pronounced in settings experiencing prolonged insecurity and health workforce displacement. Similar evidence from fragile states has shown that emergency obstetric care capacity is highly sensitive to healthcare workforce instability and supply chain disruption [16, 38]. Importantly, the findings suggest that maternal and newborn mortality in humanitarian settings is not solely attributable to increased disease burden or obstetric complications themselves but also to systemic failures in emergency referral systems, transportation mechanisms, and timely clinical intervention capacity [13].

Delays in accessing emergency care were repeatedly identified as major contributors to maternal hemorrhage related deaths, obstructed labor complications, hypertensive emergencies, neonatal asphyxia, and preventable stillbirths [13, 16]. These findings reinforce the central role of emergency obstetric and newborn care strengthening within humanitarian response frameworks. Another major finding of this umbrella review concerns the widespread deterioration of respectful maternity care during humanitarian crises. Women frequently experienced verbal abuse, neglect, poor communication, lack of privacy, discrimination, and absence of informed consent during childbirth [13, 14, 15, 38]. Similar evidence from Sub Saharan Africa has demonstrated that disrespect and abuse during childbirth remain widespread even outside humanitarian settings, although crisis conditions appear to intensify these challenges [15, 37]. The worsening of respectful maternity care during humanitarian emergencies may be explained by multiple interacting factors, including severe workforce shortages, provider burnout, overcrowded facilities, psychological stress, lack of supervision, and weakened accountability systems [16, 38].

In addition, humanitarian settings frequently involve culturally diverse displaced populations, language barriers, and social marginalization, which may further complicate patient provider interactions [17]. Importantly, several reviews suggested that disrespectful maternity care contributed to delayed healthcare seeking and mistrust toward health facilities, particularly among adolescents and displaced women [14, 15, 38]. These findings demonstrate that respectful maternity care should be considered a core humanitarian health priority rather than a secondary quality indicator.

The review also identified profound inequities in maternal and newborn healthcare access across humanitarian settings. Refugees, internally displaced persons, rural populations, adolescents, and socioeconomically disadvantaged women consistently experienced greater barriers to healthcare utilization [13, 14, 15, 17, 18]. However, the degree of inequity differed across settings. Some reviews reported relatively improved healthcare access in organized refugee camp settings supported by humanitarian agencies, whereas women living outside formal camp systems frequently experienced severe exclusion from healthcare services [19, 20] (evidence from Syrian refugees in Lebanon, outside Sub‐Saharan Africa). Geographic isolation, insecurity, transportation barriers, and financial hardship were consistently identified as major determinants of inequitable healthcare access [13, 14, 17].

Similar evidence from humanitarian settings has demonstrated that crises often amplify pre‐existing social and economic inequalities because vulnerable populations lose access to transportation, income, social protection systems, and healthcare simultaneously [7]. Gender inequality and limited female decision making power additionally reduced healthcare seeking behavior in several settings, particularly in conservative and conflict affected regions [49]. The findings therefore emphasize that improving healthcare quality in humanitarian settings requires both health system strengthening and broader social protection strategies targeting vulnerable populations.

Weak health system readiness emerged as one of the most consistently reported determinants of poor maternal and newborn healthcare quality. Severe shortages of midwives, obstetricians, anesthetists, neonatal nurses, and community health workers were reported across most humanitarian settings [8, 9, 10, 11, 12, 13, 14, 38]. Reviews involving prolonged conflict settings reported particularly severe workforce attrition resulting from insecurity, migration, and attacks on healthcare infrastructure [42]. Frequent stock outs of oxytocin, magnesium sulfate, antibiotics, blood products, and neonatal medications further compromised healthcare delivery [10, 13, 16]. Nevertheless, important contextual differences were observed across reviews. Some humanitarian settings with strong donor engagement and coordinated humanitarian response mechanisms demonstrated relatively better continuity of maternal health services despite ongoing crises [8, 9, 38]. This finding suggests that healthcare quality deterioration during humanitarian emergencies is partially preventable when resilient healthcare systems, coordinated humanitarian governance, and sustainable financing mechanisms are maintained.

Importantly, this umbrella review identified several interventions associated with improved maternal and newborn healthcare quality in fragile settings. Community health worker programs, mobile clinics, outreach services, and task shifting interventions improved healthcare accessibility and continuity of care among displaced populations [8, 9, 10, 18]. Similar evidence has shown that decentralized community based healthcare models are particularly valuable in humanitarian settings where fixed healthcare infrastructure may be inaccessible or destroyed [9, 11]. However, the effectiveness of these interventions appeared to vary across settings depending on security conditions, funding sustainability, healthcare workforce training, and integration with formal health systems [11, 12]. Reviews also suggested that respectful maternity care training, culturally sensitive healthcare approaches, and community engagement strategies improved patient experiences and trust toward healthcare systems [8, 9, 15]. These findings indicate that quality improvement interventions in humanitarian settings should combine both clinical capacity strengthening and patient centered healthcare approaches.

This umbrella review additionally identified substantial methodological and evidence related gaps within the existing literature. Considerable heterogeneity existed across reviews regarding healthcare quality definitions, outcome indicators, humanitarian context classification, and synthesis methods [12, 38]. Several reviews combined evidence from conflict settings, refugee camps, epidemic affected regions, and fragile health systems despite substantial contextual differences between these humanitarian environments [9, 12]. Such heterogeneity limits direct comparability across studies and complicates interpretation of pooled findings. Furthermore, evidence from highly insecure regions remained limited because operational and ethical challenges frequently restrict primary data collection in active conflict zones [42]. The available evidence also disproportionately focused on maternal healthcare services, whereas neonatal care quality, maternal mental health integration, continuity of care after displacement, and long term maternal and neonatal outcomes received comparatively limited attention [8, 9]. These findings highlight important priorities for future humanitarian maternal and newborn health research.

### 4.1 Proposed Framework for Research and Practice

The WHO framework describes what quality of care is, but the included reviews give little guidance on how to apply it across different crisis contexts, and variable methods limit evidence‐informed recommendations. Building on the findings above, we propose a four‐component framework for research and practice. It is a conceptual proposal derived from this synthesis and has not been tested.

First, stratify the context. Programs and studies should state the crisis type (armed conflict, displacement, epidemic, or disaster), its phase (acute or protracted), conflict intensity, and the setting (camp or non‐camp, urban or rural), because disruption and the feasibility of interventions differed across these strata [11, 12, 17].

Second, measure a minimum common set of quality indicators across the six dimensions, including respectful care and neonatal outcomes, so that findings can be compared and pooled. Heterogeneous definitions and measures currently prevent comparison [12, 38].

Third, match intervention bundles to context. Where facilities function, combine workforce training with supervision, supply chain and referral strengthening, and respectful care. Where facilities are damaged or inaccessible, prioritize community‐delivered interventions that do not depend on functional facilities, such as breastfeeding support, kangaroo mother care, and cord care, supported by community health workers and mobile or digital supervision [42].

Fourth, build learning and accountability into programs through routine process and outcome data, community feedback mechanisms, and evaluation designs, for example controlled before‐and‐after studies, that can report effects on morbidity and mortality, because most evidence to date concerns process outcomes (Table 4). Quality improvement methods have been used to strengthen health systems in resource‐poor settings [52]. Together these components move from describing deficits towards a context‐sensitive agenda for implementation research and correspond to review questions 1 to 4.

## 5 Strengths and Limitations

### 5.1 Strengths

This umbrella review provides a comprehensive synthesis of review‐level evidence regarding maternal and newborn healthcare quality in humanitarian and crisis settings across Sub‐Saharan Africa. The review incorporated evidence from multiple humanitarian contexts including armed conflict, forced displacement, fragile health systems, and refugee settings. Methodological strengths include protocol registration, duplicate screening and extraction, use of AMSTAR 2 methodological appraisal, overlap assessment, and synthesis across multiple dimensions of healthcare quality. The review additionally examined both clinical and patient‐centered dimensions of care, including effectiveness, safety, respectful maternity care, equity, continuity of care, and health system readiness.

## 6 Limitations

Several limitations should be considered when interpreting the findings of this umbrella review. The included reviews demonstrated substantial methodological heterogeneity in populations, humanitarian contexts, outcome definitions, and synthesis approaches. Two included records were assessed as low or critically low methodological quality because of incomplete risk of bias assessment, lack of protocol registration, and limited publication bias evaluation.

Moderate overlap of primary studies across systematic reviews may also have influenced evidence synthesis despite overlap management procedures. Furthermore, evidence from highly insecure settings remained underrepresented because operational and ethical challenges frequently restrict research implementation during active conflict. Publication bias may additionally affect the available evidence base because humanitarian intervention studies demonstrating positive outcomes may be more likely to be published than studies reporting null or negative findings. Incomplete documentation is a further source of bias: records in crisis settings are often missing or inconsistently kept, so the primary studies underlying the included reviews, and the reviews themselves, may under‐report service disruption, deaths, and mistreatment, and some reviews gave limited detail on search or exclusion decisions. The search began in January 2015, so earlier reviews were not considered, and only English language reports were included.

## 7 Conclusion

This umbrella review demonstrates that maternal and newborn healthcare quality in humanitarian and crisis settings across Sub‐Saharan Africa remains severely compromised across multiple dimensions of care, including effectiveness, safety, respectful maternity care, equity, continuity of care, and health system readiness.

Humanitarian crises consistently disrupted essential maternal and newborn healthcare services through insecurity, forced displacement, healthcare workforce shortages, damaged infrastructure, weak referral systems, and shortages of essential medicines and supplies. Vulnerable populations, particularly refugees, internally displaced women, adolescents, and rural communities, experienced the greatest barriers to accessing quality maternal and newborn healthcare. Despite these challenges, evidence suggests that community‐based interventions, mobile health services, task‐shifting approaches, respectful maternity care initiatives, and strengthened humanitarian coordination mechanisms can improve maternal and newborn healthcare delivery in fragile settings. In answer to the four review questions, the evidence shows (1) compromised quality across six dimensions; (2) insecurity, workforce, supply, referral, sociocultural, and economic barriers alongside community, workforce, and coordination facilitators; (3) that most strategies were evaluated on process rather than mortality outcomes; and (4) gaps in neonatal care, postnatal care, maternal mental health, long‐term neonatal outcomes, and standardized quality measurement.

## 8 Recommendations

1. Governments and humanitarian agencies should prioritize maternal and newborn healthcare within all humanitarian preparedness and response strategies.
2. Emergency obstetric and newborn care services, referral systems, and supply chain mechanisms should be strengthened in crisis affected settings.
3. Investments in skilled healthcare workforce development, particularly midwifery and neonatal care training, should be expanded to improve healthcare quality and continuity of care.
4. Humanitarian health programs should integrate respectful maternity care and culturally sensitive approaches into maternal and newborn healthcare services. Specific actions for humanitarian agencies include adopting the WHO standards for quality maternal and newborn care [14] and a respectful maternity care charter in program guidance; embedding respectful care content in emergency obstetric and newborn care training and mentorship; ensuring privacy, birth companions, informed consent, and female providers and interpreters in temporary and camp facilities; establishing community feedback and complaint mechanisms; and adding respectful care indicators to routine monitoring and to Minimum Initial Service Package implementation checks.
5. Future research should focus on standardized quality of care measurement, neonatal healthcare quality, maternal mental health, and implementation effectiveness of quality improvement interventions in humanitarian settings.

## Author Contributions

**Endale Tamiru Burayu:** conceptualization, methodology, software, data curation, formal analysis, investigation, supervision, validation, funding acquisition, resources, visualization, project administration, writing – review and editing, writing – original draft. **Zelalem Banjaw Zegeye:** methodology, conceptualization, software, validation, funding acquisition, investigation, supervision, data curation, formal analysis, resources, project administration, writing – review and editing, writing – original draft, visualization. **Gemechu Gelan Bekele:** conceptualization, methodology, software, data curation, formal analysis, visualization, investigation, supervision, project administration, writing – review and editing, resources, funding acquisition, validation, writing – original draft.

## Funding

The authors have nothing to report.

## Ethics Statement

Not applicable for this study. This umbrella review synthesized evidence from existing published systematic reviews and meta‐analyses and did not involve primary data collection from human participants.

## Conflicts of Interest

The authors declare no conflicts of interest.

## Author Statement

All authors have read and approved the final version of the manuscript. Endale Tamiru Burayu had full access to all of the data in this study and takes complete responsibility for the integrity of the data and the accuracy of the data analysis.

## Transparency Statement

The corresponding author (Endale Tamiru Burayu) affirms that this manuscript is an honest, accurate, and transparent account of the study being reported; that no important aspects of the study have been omitted; and that any discrepancies from the study as planned (and, if relevant, registered) have been explained.

## Supporting information


Supporting File 1

Supporting File 2

Supporting File 3

## Data Availability

All data generated or analyzed during this umbrella review are included in this published article and its supplementary information files.
