# Supplementary material for: Quality of Maternal and Newborn Care in Humanitarian and Crisis Settings in Sub‐Saharan Africa: An Umbrella Review
Source: Health Sci Rep. 2026 Nov 4;9(11):e73463. doi: 10.1002/hsr2.73463 (PMC13732563; doi:10.1002/hsr2.73463)
Supplement: Supplementary file 1 [file HSR2-9-e73463-s001.docx]

**Supplementary Appendix 2. AMSTAR 2 Methodological Appraisal of Included Reviews**

| **First Author, Year** | **Protocol Registered** | **Comprehensive Literature Search** | **Duplicate Screening and Extraction** | **Risk of Bias Assessment** | **Publication Bias Assessment** | **Explanation of Excluded Studies** | **Overall AMSTAR 2 Rating** |
| --- | --- | --- | --- | --- | --- | --- | --- |
| Munyuzangabo et al., 2021 | Yes | Yes | Yes | Yes | Yes | Yes | High |
| Rodo et al., 2022 | Yes | Yes | Yes | Partial | No | Yes | Moderate |
| Kampalath et al., 2022 | Partial | Yes | Yes | Yes | No | Yes | Moderate |
| McBride et al., 2022 | No | Yes | Yes | Partial | No | Yes | Moderate |
| Jordan et al., 2021 | Yes | Yes | Yes | Yes | Yes | Yes | High |
| Geleto et al., 2018 | Yes | Yes | Yes | Yes | Yes | Yes | High |
| Kyei-Nimakoh et al., 2017 | Partial | Yes | Yes | Yes | Partial | Yes | Moderate |
| Amberg et al., 2023 | Yes | Yes | Yes | Yes | Yes | Yes | High |
| Adeyanju et al., 2025 | Partial | Yes | Yes | No | No | Partial | Moderate |
| Banke-Thomas et al., 2020 | Yes | Yes | Yes | Partial | No | Yes | Moderate |
| Lassi et al., 2016 | No | Partial | Partial | Partial | No | Partial | Low |
| Bohren et al., 2015 | No | Partial | Partial | Partial | No | No | Critically low |

Note. Overall ratings follow AMSTAR 2 rules (high: no or one non-critical weakness; moderate: more than one non-critical weakness; low: one critical flaw; critically low: more than one critical flaw). Bohren et al., 2015 was rated critically low because two critical domains were not met (protocol not registered; list of excluded studies not provided). Items on meta-analysis were not applicable to reviews without quantitative pooling. Topp et al., 2021 in the earlier version is the review by Jordan et al., 2021 (Confl Health. 2021;15:7).
