# Supplementary material for: Quality of Maternal and Newborn Care in Humanitarian and Crisis Settings in Sub‐Saharan Africa: An Umbrella Review
Source: Health Sci Rep. 2026 Nov 4;9(11):e73463. doi: 10.1002/hsr2.73463 (PMC13732563; doi:10.1002/hsr2.73463)
Supplement: Supplementary file 2 [file HSR2-9-e73463-s002.docx]

**Supplementary Appendix 3. Overlap Assessment of Included Reviews**

**Citation Overlap Assessment**

Overlap of primary studies across included reviews was assessed using citation matrix methodology and corrected covered area (CCA) calculations. The corrected covered area was calculated as (N - r) / (rc - r), where N is the total number of primary study inclusions across reviews counting repeats, r is the number of unique primary studies, and c is the number of reviews (12). Interpretation followed Pieper et al.: 0 to 5% slight, 6 to 10% moderate, 11 to 15% high, above 15% very high overlap. Overall CCA = [CCA value]% (N = [value], r = [value], c = 12). Overlap was concentrated among reviews focusing on:

- emergency obstetric and newborn care,
- maternal healthcare utilization,
- respectful maternity care,
- and health system barriers in conflict settings.

The overlap primarily reflected repeated inclusion of studies conducted in:

- Somalia,
- South Sudan,
- Democratic Republic of Congo,
- Nigeria,
- and refugee or internally displaced populations.

To minimize duplication bias:

- reviews rated low or critically low on AMSTAR 2 were used as supporting evidence only, and no theme rests solely on them (see Methods),
- findings were synthesized narratively rather than quantitatively pooled,
- and duplicated findings were interpreted cautiously during evidence synthesis.

The degree of overlap, interpreted with the CCA value above, was considered unlikely to substantially distort overall conclusions.
