# Supplementary material for: Quality of Maternal and Newborn Care in Humanitarian and Crisis Settings in Sub‐Saharan Africa: An Umbrella Review
Source: Health Sci Rep. 2026 Nov 4;9(11):e73463. doi: 10.1002/hsr2.73463 (PMC13732563; doi:10.1002/hsr2.73463)
Supplement: Supplementary file 3 [file HSR2-9-e73463-s003.docx]

**Supplementary Appendix 1. Detailed Electronic Search Strategy**

These strategies are reported in line with PRISMA 2020 item 7 and PRISMA-S. Each numbered section below gives the full, database-specific string, including CINAHL (section 5) and the Cochrane Library (section 8). Results from all sources were combined and de-duplicated before screening (1,284 records identified; 392 duplicates removed).

**Databases Searched**

- PubMed/MEDLINE
- Scopus
- Web of Science
- Embase
- CINAHL
- PsycINFO
- Global Health
- Cochrane Database of Systematic Reviews
- Joanna Briggs Institute Evidence Synthesis

**Grey Literature Sources**

- Google Scholar
- WHO Institutional Repository for Information Sharing (IRIS)
- ReliefWeb
- UNICEF repositories
- UNFPA repositories
- Humanitarian organization websites

**Search Limits**

- Publication period: January 1, 2015 to March 31, 2026
- Language: English only

# 1. PubMed/MEDLINE Search Strategy

((“maternal health services”[Mesh] OR “maternal care” OR “maternal healthcare” OR “newborn care” OR “neonatal care” OR “obstetric care” OR “emergency obstetric care” OR “postnatal care”))
AND
((“quality of care” OR “healthcare quality” OR “respectful maternity care” OR “patient-centered care” OR “health system quality” OR “continuity of care” OR safety OR effectiveness OR equity))
AND
((“humanitarian setting” OR “conflict affected” OR “armed conflict” OR “fragile setting” OR refugee OR “internally displaced persons” OR “humanitarian crisis” OR “disaster setting”))
AND
((“Sub-Saharan Africa” OR “Africa South of the Sahara”[Mesh] OR Ethiopia OR Somalia OR Sudan OR “South Sudan” OR Nigeria OR “Democratic Republic of Congo”))
AND
((“systematic review” OR “meta-analysis” OR “review synthesis” OR “scoping review”))
Filters: English; Publication date from 2015/01/01 to 2026/03/31

# 2. Scopus Search Strategy

TITLE-ABS-KEY(
(
“maternal care” OR
“maternal healthcare” OR
“newborn care” OR
“neonatal care” OR
“obstetric care” OR
“emergency obstetric care” OR
“postnatal care”
)
AND
(
“quality of care” OR
“healthcare quality” OR
“respectful maternity care” OR
“patient-centered care” OR
“continuity of care” OR
safety OR effectiveness OR equity
)
AND
(
“humanitarian setting” OR
“conflict affected” OR
“armed conflict” OR
“fragile setting” OR
refugee OR
“internally displaced persons” OR
“humanitarian crisis” OR
“disaster setting”
)
AND
(
“Sub-Saharan Africa” OR
Ethiopia OR Somalia OR Sudan OR
“South Sudan” OR Nigeria OR
“Democratic Republic of Congo”
)
AND
(
“systematic review” OR
“meta-analysis” OR
“review synthesis” OR
“scoping review”
)
)
AND PUBYEAR > 2014
AND LANGUAGE(english)

# 3. Web of Science Core Collection Search Strategy

TS=(
(
“maternal care” OR
“maternal healthcare” OR
“newborn care” OR
“neonatal care” OR
“obstetric care” OR
“emergency obstetric care” OR
“postnatal care”
)
AND
(
“quality of care” OR
“healthcare quality” OR
“respectful maternity care” OR
“patient-centered care” OR
“continuity of care” OR
safety OR effectiveness OR equity
)
AND
(
“humanitarian setting” OR
“conflict affected” OR
“armed conflict” OR
“fragile setting” OR
refugee OR
“internally displaced persons” OR
“humanitarian crisis” OR
“disaster setting”
)
AND
(
“Sub-Saharan Africa” OR
Ethiopia OR Somalia OR Sudan OR
“South Sudan” OR Nigeria OR
“Democratic Republic of Congo”
)
AND
(
“systematic review” OR
“meta-analysis” OR
“review synthesis” OR
“scoping review”
)
)
Refined by:
LANGUAGE: (ENGLISH)
Timespan: 2015–2026

# 4. Embase Search Strategy

(
'maternal care'/exp OR
'maternal healthcare' OR
'newborn care' OR
'neonatal care' OR
'obstetric care' OR
'emergency obstetric care' OR
'postnatal care'
)
AND
(
'quality of care' OR
'healthcare quality' OR
'respectful maternity care' OR
'patient centered care' OR
'continuity of care' OR
safety OR effectiveness OR equity
)
AND
(
'humanitarian setting' OR
'conflict affected' OR
'armed conflict' OR
'fragile setting' OR
refugee OR
'internally displaced persons' OR
'humanitarian crisis' OR
'disaster setting'
)
AND
(
'Sub-Saharan Africa' OR
Ethiopia OR Somalia OR Sudan OR
'South Sudan' OR Nigeria OR
'Democratic Republic of Congo'
)
AND
(
'systematic review'/exp OR
'meta analysis'/exp OR
'review synthesis' OR
'scoping review'
)
AND [2015-2026]/py
AND [english]/lim

# 5. CINAHL Search Strategy

(
MH "Maternal Health Services+" OR
MH "Obstetric Care+" OR
“maternal healthcare” OR
“newborn care” OR
“neonatal care”
)
AND
(
“quality of care” OR
“respectful maternity care” OR
“patient-centered care” OR
“continuity of care” OR
safety OR effectiveness OR equity
)
AND
(
“humanitarian setting” OR
“conflict affected” OR
refugee OR
“internally displaced persons” OR
“humanitarian crisis”
)
AND
(
“Sub-Saharan Africa” OR
Ethiopia OR Somalia OR Sudan OR
“South Sudan” OR Nigeria OR
“Democratic Republic of Congo”
)
AND
(
“systematic review” OR
“meta-analysis” OR
“review synthesis”
)
Limiters:
English Language;
Published Date: 20150101–20260331

# 6. PsycINFO Search Strategy

(
“maternal healthcare” OR
“newborn care” OR
“neonatal care” OR
“obstetric care”
)
AND
(
“quality of care” OR
“respectful maternity care” OR
“patient-centered care” OR
equity OR safety
)
AND
(
“humanitarian setting” OR
“armed conflict” OR
refugee OR
“internally displaced persons”
)
AND
(
“Sub-Saharan Africa” OR
Ethiopia OR Somalia OR Sudan OR
“South Sudan”
)
AND
(
“systematic review” OR
“meta-analysis” OR
“review synthesis”
)
Limiters:
English;
2015–2026

# 7. Global Health Database Search Strategy

(
“maternal healthcare” OR
“newborn care” OR
“neonatal care” OR
“obstetric care”
)
AND
(
“quality of care” OR
“healthcare quality” OR
“respectful maternity care”
)
AND
(
“humanitarian crisis” OR
“conflict affected” OR
“fragile setting” OR
refugee
)
AND
(
“Sub-Saharan Africa” OR
Ethiopia OR Somalia OR Sudan OR
“South Sudan”
)
AND
(
“systematic review” OR
“meta-analysis”
)
Publication Years:
2015–2026
Language:
English

# 8. Cochrane Library Search Strategy

(
“maternal healthcare” OR
“newborn care” OR
“obstetric care”
)
AND
(
“quality of care” OR
“respectful maternity care”
)
AND
(
“humanitarian setting” OR
“armed conflict” OR
refugee
)
AND
(
“Sub-Saharan Africa”
)
with Publication Year from 2015 to 2026

# 9. Grey Literature Search Strategy

Grey literature searches were conducted through:

- Google Scholar,
- WHO IRIS,
- ReliefWeb,
- UNICEF repositories,
- UNFPA repositories,
- and humanitarian organization websites.

The following search combinations were used:

“maternal healthcare quality” humanitarian Sub-Saharan Africa review

“newborn care” conflict settings Africa systematic review

“respectful maternity care” refugee settings Africa

“emergency obstetric care” humanitarian crisis review

“maternal and newborn health” fragile settings Sub-Saharan Africa

Only documents meeting predefined eligibility criteria and methodological standards were included in the final synthesis.
